# Maintaining cognitive and physical health across the adult lifespan: the contribution of psychosocial factors

**DOI:** 10.1007/s10865-025-00603-9

**Published:** 2025-09-30

**Authors:** Morgan K. Taylor, Kylie A. Schiloski, Margie E. Lachman

**Affiliations:** Department of Psychology, Brandeis University, 415 South Street, Waltham, MA 02453 USA

**Keywords:** Sense of control, Purpose in life, Social support, Longevity

## Abstract

Research demonstrates that demographic (e.g., age, sex), behavioral (e.g., exercise), and lifestyle (e.g., drinking habits) factors are related to individual differences in cognition and health. Although some work has examined modifiable psychosocial factors (e.g., control beliefs, purpose in life, social contact) in relation to cognition and health, they are typically examined separately. We used data from the Midlife in the United States (MIDUS) study to investigate whether a composite measure of sense of control, purpose in life, and social support assessed at the second wave (M2) would be associated with cognition (episodic memory, executive function) and health (functional health, chronic conditions) ten years later at the third wave (M3). Participants (*N* = 2,497) ranged from 33 to 83 years old. We created a continuous composite of sense of control, purpose in life, and social support by standardizing and summing the individual factor scores. Using multiple regression, we tested the composite as a predictor of 10 year changes in episodic memory, executive function, functional health, and chronic conditions. We found that those higher on the psychosocial composite demonstrated greater maintenance of cognition and health compared to those lower on the composite, even after controlling for demographic, behavioral, and lifestyle risk and protective factors. Education moderated the effect of the composite on functional health, such that having a higher psychosocial score was more predictive of better functional health among those with lower and moderate levels of education. We discuss the value of using a psychosocial composite measure and implications for public health.

**Supplementary Information:**

The online version contains supplementary material available at 10.1007/s10865-025-00603-9.

## Introduction

While aging is typically associated with declines in cognitive (Salthouse, 2012) and physical health (Yashin et al., 2007), there is wide interindividual variability in the nature and scope of these changes (Alwin & Hofer, 2011). Numerous studies have found that demographic (i.e., sex, education) and behavioral/lifestyle (i.e., physical activity, drinking habits) factors are related to individual differences in cognition and health across the adult lifespan (Gow et al., 2012; Hamm et al., 2024; Rexroth et al., 2013; Rogers et al., 2010). While these factors are modifiable, more recent work has begun to focus on modifiable attitudes and beliefs instead (Zahodne, 2021). For example, higher sense of control in one’s life, strong social support from friends and family, and a robust purpose in life have all been shown to delay or lessen the declines in older adults’ cognition and health (Boyle et al., 2010; Raldiris et al., 2021; Vila, 2021). However, these psychosocial factors are typically examined separately from one another and can coexist to varying degrees within individuals. Here, we investigate the additive effects of three psychosocial factors (sense of control, purpose in life, and social support) on cognition (episodic memory, executive function) and health (functional health, chronic conditions) across the adult lifespan.

Control beliefs encompass the perception that one can influence what happens in their life and the extent to which their actions can bring about desired outcomes. They include beliefs and expectations about one’s abilities and perceived external constraints (Lachman, 2006). Individuals that have a higher sense of control often demonstrate better cognitive functioning (Raldiris et al., 2021). For example, middle-aged and older adults who have higher control beliefs remember more words during a recall test (Lachman & Andreoletti, 2006) and demonstrate less decline in executive functioning over ten years (Schiloski & Lachman, 2024). Similarly, individuals with a higher sense of control have been found to have better physical (e.g., fewer physical limitations and chronic conditions), mental (e.g., greater optimism and life satisfaction), and social outcomes (e.g., increased contact with friends) compared to those with a lower sense of control (Hong et al., 2021). Those who have higher control beliefs are more likely to engage in health-promoting behaviors like exercising, eating a healthy diet, and engaging in cognitively stimulating activities, which ultimately influences their later health and cognition (Lachman & Firth, 2004; Schiloski & Lachman, 2024).

Purpose in life involves the goals, intentions, and sense of direction that contribute to the feeling that life is meaningful (Ryff, 1989). Individuals that derive more meaning or purpose from their lives are better protected from cognitive decline: they are less likely to develop mild cognitive impairment and Alzheimer’s Disease and demonstrate a slower rate of perceived and actual cognitive decline compared to those who lack purpose in life (Boyle et al., 2010; Sutin et al., 2020; Wingo et al., 2020). Having a robust purpose is life is also related to lower risk of stroke (Kim et al., 2013), cardiovascular disease (Kim et al., 2019) and mortality (Alimujiang et al., 2019). Similar to control beliefs, the connection between purpose in life and physical and cognitive health outcomes appears to be related to health behaviors (e.g., physical activity, healthy sleep habits; Kim et al., 2020; Yemiscigil & Vlaev, 2021).

Social support refers to an individual’s perception of the extent to which they can count on others from their social networks when needed (Kelly et al., 2017). Individuals with strong social support often have better cognitive function, including episodic memory and executive function (Seeman et al., 2001, 2011; Zuelsdorff et al., 2017). Likewise, meta-analyses have revealed that those with greater social support have a reduced risk of disease and mortality (Holt-Lunstad et al., 2010; Vila et al., 2021). According to the stress-buffering hypothesis, social support benefits health and longevity because social bonds lessen or eliminate the adverse consequences of prolonged stress responses (i.e., cortisol production, inflammation). In other words, safety social cues from friends and loved ones induce the inhibition of stress responses and promote homeostasis throughout the body, which ultimately helps reduce the incidence of disease and mortality (Vila et al., 2021).

Individually, it is clear that these psychosocial factors are related to changes in cognition and health. However, few studies have considered such factors together to examine their additive or combined effects (see Lachman & Agrigoroaei, 2010; Agrigoroaei & Lachman, 2011). This type of multidimensional approach has become more common in recent years. For example, one study found that those with a greater number of healthy lifestyle factors (i.e., low alcohol consumption, balanced diet) had a lower risk of mortality than those with fewer (Loef & Walach, 2012). Other studies have found that maintaining high levels of multiple facets of well-being (e.g., lifestyle, SES, psychosocial) is associated with a lower risk of dementia (Zhang et al., 2023) and cardiometabolic disease (Guimond et al., 2022). Furthermore, some work has found that exposure to a higher number of favorable psychosocial factors in childhood (e.g., social adjustment, positive family environment) is associated with better cardiovascular health in adulthood and the effects are additive (Appleton et al., 2013; Pulkki-Raback et al., 2015). These studies highlight the value in moving beyond a single factor approach to investigate how multiple indicators may jointly relate to cognition and health. This is critical because these factors do not exist in isolation. Rather, they tend to occur in combination with each other within individuals (Guimond et al., 2022). For example, dementia risk scores and measures of allostatic load are composites that comprise of several health indicators and may predict disease and mortality better than individual markers (Anstey et al., 2022; Seeman et al., 2004). Thus, we take a similar approach in order to identify the additive effect of these psychosocial factors on long-term cognition and health.

In the current work, we examine the combined contribution of sense of control, purpose in life, and social support on longitudinal changes in cognition and health and consider their protective effect over and above the influence of demographic, behavioral, and lifestyle risk and protective factors. We chose these psychosocial factors because they are consistently found to be important for cognition and health (Alimujiang et al., 2019; Boyle et al., 2010; Zahodne, 2021) and are amenable to change via intervention (Lachman et al., 2015; Shin & Steger, 2014). They also relate to stress reduction, an important factor of maintaining good cognitive and physical health (Sutin et al., 2024; Vila et al., 2021). Our cognitive outcomes consisted of tests that probe episodic memory and executive function, two major dimensions of cognition that have been shown to be related to the psychosocial factors (Raldiris et al., 2021; Sutin et al., 2020; Seeman et al., 2001). Our health outcomes consisted of self-reported functional health and total number of chronic conditions, two commonly used measures that are good representations of different aspects of health (Koroukian et al., 2016) and relate to the psychosocial factors (Hong et al., 2021; Kim et al., 2019, Vila et al., 2021). Investigating these cognitive and health outcomes together allows us to jointly assess two important capacities that decline in older adulthood and identify protective factors that may help individuals maintain both as they age.

A recent related study found that individuals with higher levels of these three psychosocial factors had better health outcomes and less chronic inflammation than those with lower levels of the factors (Lachman & Schiloski, 2024). We extend this work by 1) using a larger and more representative sample, 2) investigating cognition in addition to health, 3) analyzing longitudinal changes in outcomes after 10 years, and 4) using an additive composite instead of a latent factor approach. We hypothesized that a composite measure of sense of control, purpose in life, and social support would predict ten year changes in episodic memory, executive function, functional health, and chronic conditions, such that those who scored higher on the psychosocial composite would demonstrate greater maintenance of cognition and health than those who scored lower on the composite. Furthermore, given sociodemographic differences in cognition and health (Rexroth et al., 2013; Yashin et al., 2007; Davies et al., 2018), we investigated age, sex and education as moderators of these relationships.

## Method

### Participants

Data were drawn from the second (M2: 2004–2005) and third (M3: 2013–2014) waves of the Midlife in the United States (MIDUS) study, a national longitudinal study that investigates the biopsychosocial factors that influence the cognitive, physical, and mental health of individuals across the adult lifespan (Radler & Ryff, 2010). Core participants were recruited via random digit dialing across the United States and enrolled individuals completed telephone interviews and self-administered questionnaires. To increase diversity, a subsample of African American participants was recruited from Milwaukee, Wisconsin. Using area probability sampling methods, participants were selected from areas with high concentrations of African Americans (based on the 2000 census). The sampling was stratified by age, gender, and SES. These individuals were interviewed in their homes using a computer assisted personal interview protocol and later completed self-administered questionnaires. All measures paralleled those used in the larger core sample. Complete details of the MIDUS study are available at www.midus.wisc.edu. Participants were included in our sample if they had sense of control, purpose in life, and social support data at M2 and demographic, lifestyle, and behavioral risk/protective factors and cognitive and health outcome data at M2 and M3. Thus, our analysis sample consisted of 2,497 individuals (M2 *M*_age_ = 54.9, range = 33–83, 88% White, 57% women; see Table 1). Participants that did not have all variables and covariates of interest at M2 and M3 (*n* = 3,059) were excluded from analysis (see Supplemental Fig. 1). This excluded sample included more men and non-White individuals who were less educated and had worse cognitive and health outcomes on average (see Supplemental Table 1). As this work is an extension of Lachman and Schiloski (2024), which only used the MIDUS biomarker subsample, there is some overlap of participants (885 out of 2,497), psychosocial measures (sense of control, purpose in life, social support), and two health variables (functional health, chronic conditions). The MIDUS study was approved by the University of Wisconsin-Madison Institutional Review Board (Protocol # 2016–1051).

**Table 1:** Participant characteristics

Variables	Mean (*SD*)
M2 Age	54.85 (11.22)
M2 Sex	57% women, 43% men
M2 Race	88% White, 12% non-White
M2 Years of Education	14.50 (2.65)
M2 Episodic Memory	.129 (.964)
M3 Episodic Memory	− .030 (.992)*
M2 Executive Function	.184 (.930)
M3 Executive Function	− .180 (.749)*
M2 Functional Health	83.86 (23.5)
M3 Functional Health	75.37 (28.5)*
M2 Chronic Conditions	2.60 (2.57)
M3 Chronic Conditions	3.29 (2.78)*

N = 2,497. Functional Health ranged from 0 to 100. Chronic Conditions ranged from 0 to 30. *There was a significant decrease in episodic memory, executive function, and functional health, and significant increase in chronic conditions between M2 and M3

**Fig. 1:**
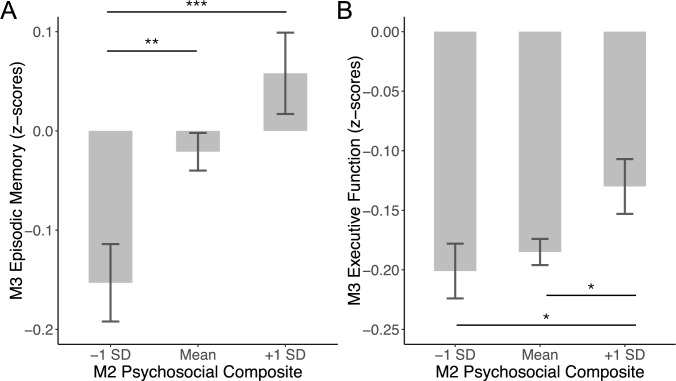
Cognition Results *Note*: Episodic memory (**A**) and executive function (**B**) results at M3 controlling for demographic, behavioral, and lifestyle covariates and M2 episodic memory and executive function, respectively. For visualization purposes, we divided participants based on whether their psychosocial composite was at the mean or one standard deviation above or below it. Participants higher on the psychosocial composite (i.e., + 1 SD) experienced greater maintenance of cognition compared to those lower on the composite. *p < .05. ** p < .01. *** *p* < .001

## Measures

### Psychosocial Composite

The psychosocial composite is a continuous measure derived from M2 measures of sense of control, purpose in life, and social support. We computed z-scores of each psychosocial factor and summed the z-scores together (*M* = 0.0, *SD* = 2.40, range = − 10.10 – 4.07, α = 0.72).

### M2 Sense of Control

This measure was assessed with 12 items from the MIDUS Sense of Control Scale (Lachman & Weaver, 1998). The score was calculated by averaging two subscales that measured personal mastery (i.e., “I can do just about anything I really set my mind to”) and perceived constraints (i.e., “What happens in my life is often out of my control”). The scores were assessed with a Likert scale that ranged from 1 (strongly agree) to 7 (strongly disagree). Scores were reverse coded for personal mastery such that higher scores indicated a higher sense of control. The sense of control measure demonstrated good internal consistency (α = 0.82).

### M2 Purpose in Life

This measure consisted of the average score from 7 items of the Ryff Scales of Psychological Well-Being (Ryff et al., 1995). Sample items included statements like, “I sometimes feel as if I’ve done all there is to do in life.” The Likert scale ranged from 1 (strongly agree) to 7 (strongly disagree) and positively worded items were reverse coded, so higher scores indicated greater purpose in life. The purpose in life measure demonstrated acceptable internal consistency (α = 0.69).

### M2 Social Support

This measure comprised of the average of 4 items that assessed the quality of social support one receives from three different groups: family, friends, and spouses/partners (Walen & Lachman, 2000). Sample items included statements like, “How much do they understand the way you feel?” and “How much can you rely on them for help if you have a serious problem?”. The scale ranged from 1 (never) to 4 (often), so higher scores indicated higher quality of social support. The social support measure demonstrated good internal consistency (α = 0.87).

### Cognition

Cognition was measured at M2 and M3. Seven cognitive dimensions were tested using the Brief Test of Adult Cognition by Telephone (BTACT; Lachman & Tun, 2008). Two measured episodic memory (immediate and delayed recall of 15 words), while the other five measured different aspects of executive function. Tasks included backwards digit span (working memory), category verbal fluency (fluency), number series (inductive reasoning), backwards counting (processing speed), and the Stop and Go Switch Task (attention switching and inhibitory control). Two cognitive factors were computed following confirmatory factor analysis (see Lachman et al., 2010): episodic memory (immediate and delayed word recall) and executive functioning (all other tests). The two cognitive factor scores were computed as means of the z-scored tests loading on to the factors at M2. The M3 tests were standardized using the means and standard deviations from M2 to allow for examination of change.

### Functional Health

Functional health was measured at M2 and M3 using seven items from the Physical Functioning subscale of the SF-36 Health Survey (Stewart et al., 1992). Participants were given a list of everyday activities—climbing stairs, carrying groceries, bathing/dressing, bending/kneeling, walking more than one mile, walking one block, and walking several blocks—and had to rate how much their health limited them in those activities on a scale from 1 (a lot) to 4 (not at all). This measure demonstrated high internal consistency (α = 0.92). We averaged participants’ ratings of each activity to make one functional health score and transformed these values so that they ranged from 0 to 100 (as suggested in Stewart et al., 1992), such that higher numbers indicate greater functional health.

### Chronic Conditions

Chronic conditions were measured at M2 and M3. Participants were asked whether they experienced any of 30 possible conditions, including asthma, thyroid disease, stomach trouble, sleep problems, and high blood pressure. Responses were coded as “no” = 0 and “yes” = 1. Scores ranged from 0 to 30, such that higher numbers indicate a greater number of chronic conditions.

### Covariates

We included M2 demographic (age, sex, race, education) and behavioral/lifestyle (smoking, alcohol/drug problems, waist circumference, physical activity) factors as covariates in all our models because they relate to the study predictors and outcomes and we wanted examine the effects of the psychosocial factors over and above these established risk and protective factors (see Supplemental Table 2). For smoking, participants were asked (1) if they have ever smoked cigarettes regularly and (2) if they currently smoke cigarettes regularly. Current smokers were given a 1, while participants who have never smoked regularly or do not currently smoke were given a 2. For alcohol/drug problems, participants were asked if they had experienced or been treated for alcohol and drug problems in the last 12 months. Participants who responded “No” were given a 0, while those who responded “Yes” were given a 1. Waist circumference was used as an indicator for obesity and was measured in inches around the navel and standardized for men and women. For physical activity, participants reported the frequency of engaging in vigorous physical activities (e.g., running, lifting heavy objects) during the summer and winter months. The total physical activity score consisted of the average of summer and winter ratings, which ranged from 1 (never) to 6 (several times a week or more).

**Table 2:** Correlations between key study variables

	1	2	3	4	5	6	7	8	9	10
Factors	*r*	*r*	*r*	*r*	*r*	*r*	*r*	*r*	*r*	*r*
1. M2 Psychosocial Composite	–									
2. M2 Age	.050*	–								
3. M2 Sex	− .008	− .014	–							
4. M2 Education	.154***	− .117***	− .133***	–						
5. M3 Episodic Memory	.105***	− .368***	.243***	.173***	–					
6. M3 Executive Function	.122***	− .444***	− .108***	.373***	.424***	–				
7. M3 Functional Health	.223***	− .318***	− .131***	.252***	.246***	.333***	–			
8. M3 Chronic Conditions	− .226***	.227***	.152***	− .174***	− .180***	− .279***	− .543***	–		
9. M2 Sense of Control	.821***	.021	− .094***	.148***	.078***	.130***	.234***	− .246***	–	
10. M2 Purpose in Life	.831***	.006	.003	.156***	.107***	.112***	.198***	− .174***	.587***	–
11. M2 Social Support	.748***	.094***	.070***	.066***	.065***	.049*	.105***	− .123***	.387***	.410***

* *p* < .05. **p < .01.*** *p* < .001

We also included M2 episodic memory, M2 executive function, M2 functional health, and M2 chronic conditions as covariates in respective models in order to establish a baseline and measure residual change in those cognitive and health outcomes.

### Statistical Analysis

Analyses were performed using SPSS (IBM Corp, 2023). To correct for multiple comparisons, we performed a Bonferroni test and adjusted the p-value accordingly. An α of .0125 was used as a threshold for statistical significance in all analyses (i.e., *p* < .0125). Multiple linear regression was used to assess the relationship between the psychosocial composite and cognitive/health outcomes longitudinally. M3 episodic memory, M3 executive function, M3 functional health, and M3 chronic conditions were outcome variables and the psychosocial composite and demographic and behavioral/lifestyle covariates were predictors in respective models. Baseline cognition/health outcomes were also predictors in respective models to examine residual change. Age, sex, and education were entered as interaction terms in separate models. Predictors and moderating variables were mean centered prior to analysis.

## Results

### Descriptive Statistics

Table 1 summarizes sample demographics and descriptive statistics, while Table 2 provides correlations for key variables. On average, episodic memory decreased from .129 to − .030 from M2 to M3, *t*(2496) = 8.43, *p* < .001, 95% CI = [.123, .197], and executive function decreased from .184 to − .180 over the same duration, *t*(2496) = 30.63, *p* < .001, 95% CI = [.340, .387]. Functional health decreased from 84 to 75 from M2 to M3, *t*(2391) = 18.35, *p* < .001, 95% CI = [7.52, 9.32], and chronic conditions increased from 2.6 to 3.3 over the same time period, *t*(2355) =  − 14.90, *p* < .001, 95% CI = [− .781, − .599].

### Episodic Memory

Linear regression revealed that the psychosocial composite was positively associated with episodic memory performance, *b* = .027, *SE* = .007, *β* = .064, *t* = 3.93, *p* < .001 (see Table 3). In other words, participants higher on the composite showed greater maintenance of episodic memory over 10 years compared to those lower on the composite, after controlling for baseline episodic memory and demographic (age, sex, race, education) and behavioral/lifestyle (smoking, alcohol/drug problems, waist circumference, physical activity) variables (see Fig. 1A). In separate models, we found that age (*b* = .001, *SE* = .001, *t* = .188, *p* = .060), sex (*b* = .020, *SE* = .013, *t* = 1.48, *p* = .140), and education (*b* = .003, *SE* = .003, *t* = 1.23, *p* = .260) did not moderate the relationship between the composite and episodic memory. Thus, having a higher score on the psychosocial composite was beneficial for all participants, regardless of their age, sex, and level of education.

**Table 3:** Regression models for M3 cognition and health outcomes

	Model 1	Model 2	Model 3	Model 4
	M3 Episodic Memory	M3 Executive Function	M3 Functional Health	M3 Chronic Conditions
Predictors	*b* (*SE*), *β*	*b* (*SE*), *β*	*b* (*SE*), *β*	*b* (*SE*), *β*
(Intercept)	.182 (.191)	.443 (.110)***	37.26 (5.39)***	1.09 (.514)
M2 Psychosocial Composite	.027 (.007), .064***	.010 (.004), .032**	.892 (.183), .075***	− .053 (.019), − .046**
M2 Age	− .024 (.001), − .268***	− .015 (.001), − .222***	− .529 (.040), − .207***	.026 (.004), .105***
M2 Sex	.322 (.033), .161***	− .030 (.019), − .020	− 2.72 (.869), − .047**	.293 (.088), .052***
M2 Education	.024 (.006), .065***	.014 (.004), .049***	.653 (.168), .061***	− .040 (.017), − .038
M2 Race	− .105 (.050), − .034	− .120 (.029), − .052***	− .264 (1.32), − .003	.390 (.134), .045**
M2 Smoking	.098 (.047), .034	.044 (.027), .020	6.17 (1.27), .074***	− .376 (.128), − .046**
M2 Alcohol/Drug	.185 (.159), .019	− .210 (.091), − .028	− .570 (4.32), − .002	− 1.67 (.448), − .058***
M2 Waist Circumference	− .022 (.017), − .021	.002 (.010), .003	− 4.31 (.480), − .142***	.271 (.047), .092***
M2 Physical Activity	.032 (.021), .025	− .001 (.012), − .001	− .040 (.555), − .001	− .142 (.056), − .039**
M2 Episodic Memory	.419 (.018), .407***	–	–	–
M2 Executive Function	–	.527 (.012), .654***	–	–
M2 Functional Health	–	–	.606 (.021), .502***	–
M2 Chronic Conditions	–	–	–	.632 (.019), .575***

M2 = MIDUS 2. M3 = MIDUS 3. *b* = unstandardized coefficient. *SE* = standard error. *β* = standardized coefficient. Significant regressions are bolded. ** p < .01. *** *p* < .001

### Executive Function

Linear regression demonstrated that the psychosocial composite was positively associated with executive functioning, *b* = .010, *SE* = .004, *β* = .032, *t* = 2.58, *p* = .010 (see Table 3). Thus, participants higher on the composite showed greater maintenance of executive function over a decade compared to those lower on the composite after controlling for baseline executive function and demographic and behavioral/lifestyle variables (see Fig. 1B). In separate models, we found that age (*b* = .0001, *SE* = .0004, *t* = .381, *p* = .704), sex (*b* = .004, *SE* = .008, *t* = .544, *p* = .586), and education (*b* = − .0003, *SE* = .001, *t* = − .201, *p* = .841) did not moderate the relationship between the composite and executive function, demonstrating the value of the composite for all participants, regardless of their age, sex, and education level.

### Functional Health

**Fig. 2:**
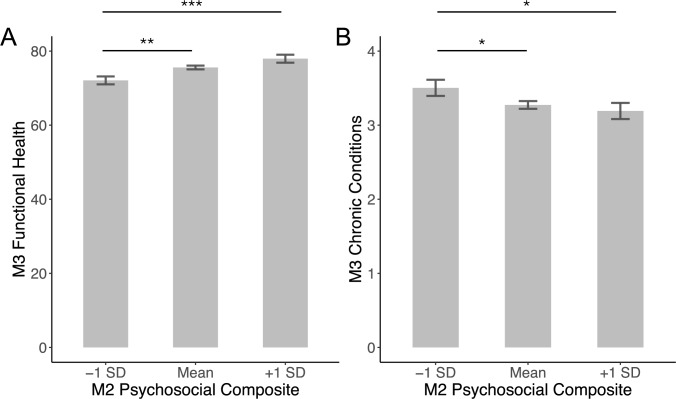
Health Results *Note*: Functional health (**A**) and chronic condition (**B**) results at M3 controlling for demographic, behavioral, and lifestyle covariates and M2 functional health and chronic conditions, respectively. For visualization purposes, we divided participants based on whether their psychosocial composite was at the mean or one standard deviation above or below it. Participants higher on the psychosocial composite (i.e., + 1 SD) experienced greater maintenance of health compared to those lower on the composite. *p < .05. ** p < .01. *** *p* < .001

**Fig. 3:**
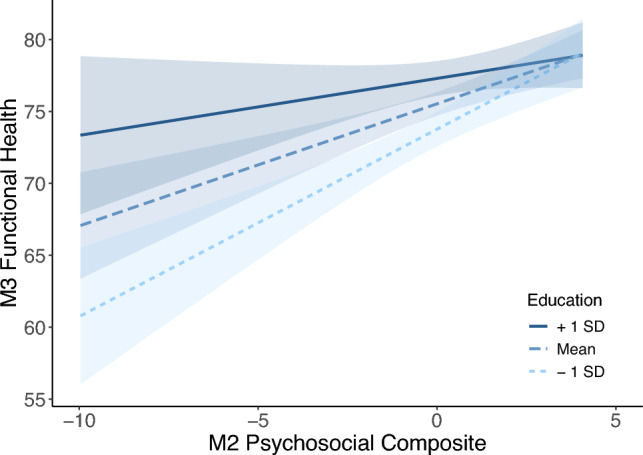
Education by Psychosocial Composite Interaction *Note*: Interaction plot showing the moderating effect of education on M3 functional health (controlling for baseline functional health). Lines represent low (1 SD below the mean), moderate (mean), and high (1 SD above the mean) levels of education with 95% confidence bands. There was a significant interaction, such that a having a higher psychosocial composite score was more beneficial for those with lower and moderate levels of education

Linear regression revealed that the psychosocial composite was positively associated with functional health, *b* = .892, *SE* = .183, *β* = .075, *t* = 4.86, *p* < .001 (see Table 3). In other words, participants higher on the composite experienced greater maintenance of functional health over 10 years compared to those lower on the composite after controlling for baseline functional health and demographic and behavioral/lifestyle variables (see Fig. 2A). In separate models, we found that age (*b* = .010, *SE* = .016, *t* = .617, *p* = .537) and sex (*b* = .053, *SE* = .356, *t* = .150, *p* = .881) did not moderate the relationship between the composite and functional health. However, there was a significant education by composite interaction (*b* = − .171, *SE* = .066, *t* = − .259, *p* = .0098). We used simple slopes analysis to probe this interaction and found that the relationship was only significant for those with lower (*b* = 1.30, *p* < .001, 95% CI = [0.83, 1.78]) and moderate (*b* = 0.85, *p* < .001, 95% CI = [0.49, 1.21]) levels of education, but not higher (*b* = 0.40, *p* = .140, 95% CI = [− 0.12, 0.92]) levels of education (see Fig. 3). Thus, being higher on the psychosocial composite was more predictive of health in those with lower and moderate educational attainment; the functional health of these individuals was more comparable to those with higher educational attainment.

### Chronic Conditions

Linear regression demonstrated that the psychosocial composite was negatively associated with number of chronic conditions, *b* = − .053, *SE* = .019, *β* = − .046, *t* = − 2.81, *p* = .005 (see Table 3). Thus, participants higher on the composite experienced greater maintenance (or less increase) of chronic conditions over a decade compared to those lower on the composite after controlling for baseline chronic conditions and demographic and behavioral/lifestyle variables (see Fig. 2B). In separate models, we found that age (*b* = − .001, *SE* = .002, *t* = .653, *p* = .514), sex (*b* = .070, *SE* = .036, *t* = 1.93, *p* = .054), and education (*b* = .007, *SE* = .007, *t* = 1.09, *p* = .276) did not moderate the relationship between the composite and chronic conditions. Thus, having a higher score on the psychosocial composite was beneficial for all participants, regardless of their age, sex, and education level.

### Individual psychosocial factors vs. psychosocial composite

Sense of control, purpose in life, and social support are all significantly correlated (see Table 2) and each factor predicts cognition and health when entered into separate regression models (see Supplemental Table 3). This is consistent with past research (Raldiris et al., 2021; Sutin et al., 2020; Hong et al., 2021; Kim et al., 2019). However, when the factors are entered simultaneously to examine net effects in one model: 1) none of them predict episodic memory, executive function, or functional health; and 2) only sense of control predicts chronic conditions (see Supplemental Table 4). Since the psychosocial factors are intercorrelated, given the overlapping variance, the net effects were no longer significant in the simultaneous regression models. Thus, our composite approach preserved each factor’s effect and reflects the combined contribution of sense of control, purpose in life, and social support on cognition and health across the adult lifespan.

## Discussion

In the current study, we investigated whether a composite of three different psychosocial factors—sense of control, purpose in life, and social support—would predict changes in cognition and health over a ten year period. We found that the psychosocial composite predicted changes in episodic memory, executive function, functional health, and chronic conditions, such that participants higher on the composite experienced greater maintenance (or less decline) in cognition and health compared to participants lower on the composite. Furthermore, education interacted with the relationship between the composite and functional health, such that having a higher psychosocial composite score was more predictive of better functional health among those with lower and moderate levels of education.

This work contributes to a growing number of studies that use composite measures to examine the combined effect of multiple factors on future cognitive and physical health (Agrigoroaei & Lachman, 2011; Guimond et al., 2022; Pulkki-Raback et al., 2015; Zhang et al., 2023). Like risk scores or measures of allostatic load, composites identify multiple related components and can address numerous outcomes simultaneously, instead of just one at a time. Furthermore, this approach is ideal because it is parsimonious and reduces measurement error—many psychosocial factors have some degree of overlap or intercorrelation, so including them simultaneously into a model often obscures their relationship with the outcome variables (Evans et al., 2013). For example, Lachman and Schiloski (2024) found that sense of control, purpose in life, and social support each predicted health when entered into a regression model individually, but only sense of control predicted health when entered simultaneously with the other factors. We found similar results with our data as well. Ultimately, the composite approach allows us to effectively test the additive effects of multiple psychosocial factors on an individual’s long-term cognition and health.

How do the effects of the psychosocial composite compare to the effects of the demographic, behavioral, and lifestyle factors included in the models? For episodic memory, age, sex, and baseline memory were the most important predictors of performance. However, the effect size of the composite was equivalent to that of education. This is surprising given the role education level plays in cognitive reserve and memory maintenance in general (Le Carret et al., 2003). This finding suggests that having high levels of sense of control, purpose in life, and social support could be just as important as education when it comes to memory performance. For health, age, waist circumference, and baseline health were among the most important predictors of functional health and chronic conditions. Yet, the effect size of the composite was analogous to that of smoking. Smoking is a risk factor that exerts considerable influence on functional health and chronic conditions (Laaksonen et al., 2006; Lebowitz, 1977). Given that the composite’s effect is equivalent to that of smoking and is greater than that of sex and education in functional health and physical activity in chronic conditions, these results demonstrate just how critical positive psychosocial factors are in terms of physical health.

We found that having a higher psychosocial composite score was more important for the functional health of those with lower and moderate levels of education. This is consistent with past work that shows that sense of control reduces mortality risk for individuals with low, but not high, educational attainment (Turiano et al., 2014). Education level has been shown to have a large influence on health. For example, those with higher levels of education experience better mental and physical health and lower rates of disability and mortality compared to those with lower levels of education (Ross & Wu, 1995). This association can be attributed to various socioeconomic and lifestyle factors; individuals with lower educational attainment are less likely to have a job or health insurance, and smoking and physical inactivity is more prevalent among this group (Woolf & Braverman, 2011). As higher education is a protective factor in itself, the additional benefit of a high psychosocial composite is likely limited among those with high educational attainment. The compensatory leveling hypothesis suggests that the health benefits of a college degree would be minimal for the most advantaged. Instead, those who are disadvantaged are the ones who stand the most to gain (Schafer et al., 2013). This likely explains why the functional health of those with a lower and moderate levels of education but a high composite score looked more similar to those with a higher education; without the intrinsic benefits of a higher education, the psychosocial composite was an important predictor of better functional health and individuals with lower and moderate education levels had more room for improvement.

Despite its strengths, our study has some limitations to note. First, our sample lacked diversity with 88% of participants being White. Thus, we cannot evaluate if we would see the same pattern of results with a more diverse sample. Next, although we focused on sense of control, purpose in life, and social support in this study, other psychosocial factors, such as optimism, positive affect, or religious involvement, have been shown to relate to cognition and health as well (Hittner et al., 2020; Rasmussen et al., 2009; Zahodne, 2021). Future work should investigate which combination of psychosocial factors is most effective in reducing the rate of cognitive and physical decline. Additionally, our results cannot speak to causation or directionality. It is possible that having better cognition and health could lead to higher levels of these psychosocial factors. For example, people with better functional health may have a stronger sense of control over their life. Future work should explore the directionality of these relationships. Finally, there is the issue of selective attrition. Individuals with poorer cognition and health dropped out of the study after M2. This ultimately limits the generalizability of our results.

Overall, we found that those higher on the psychosocial composite had better cognition and health outcomes ten years later, even after controlling for their initial level of functioning and well-established risk and protective factors, and education moderated the effect of the composite on functional health. These findings have important implications for public health. Many health recommendations and interventions focus on changing health behaviors, like engaging in more physical activity, decreasing alcohol consumption, or quitting smoking. However, some interventions may suffer from low adherence because they do not address the psychosocial factors that provide the motivation and incentive to engage in these health promoting behaviors (Middleton et al., 2013). Our work demonstrates the value and importance of considering multiple psychosocial factors together; the combined effect of sense of control, purpose in life, and social support is greater than or equivalent to that of several demographic, behavioral, and lifestyle factors. Furthermore, if different psychosocial factors relate to different outcomes, then it would be beneficial to intervene on the combination of the factors, so multiple outcomes could be addressed at once. Thus, interventions that focus on a combination of these psychosocial factors in addition to behavioral/lifestyle factors (Ngandu et al., 2015; Small et al., 2006) could be most effective in reducing the rate of cognitive and physical decline in middle and older adulthood.

## Supplementary Information

Below is the link to the electronic supplementary material.

Supplementary file1 (PDF 266 kb)

## Data Availability

The MIDUS data used in this study is available at https://midus.colectica.org/ and https://www.icpsr.umich.edu/web/ICPSR/series/203
