# Supplementary material for: Maintaining cognitive and physical health across the adult lifespan: the contribution of psychosocial factors
Source: J Behav Med. 2025 Sep 30;48(6):929–40. doi: 10.1007/s10865-025-00603-9 (PMC12494166; doi:10.1007/s10865-025-00603-9)
Supplement: Supplementary file 1 — Supplementary file1 (PDF 266 kb) [file 10865_2025_603_MOESM1_ESM.pdf]

Supplement for *Maintaining Cognitive and Physical Health Across the Adult Lifespan: The Contribution of Psychosocial Factors*

Supplemental Figure 1

Flow Diagram of Analysis Sample and Excluded Sample Participants

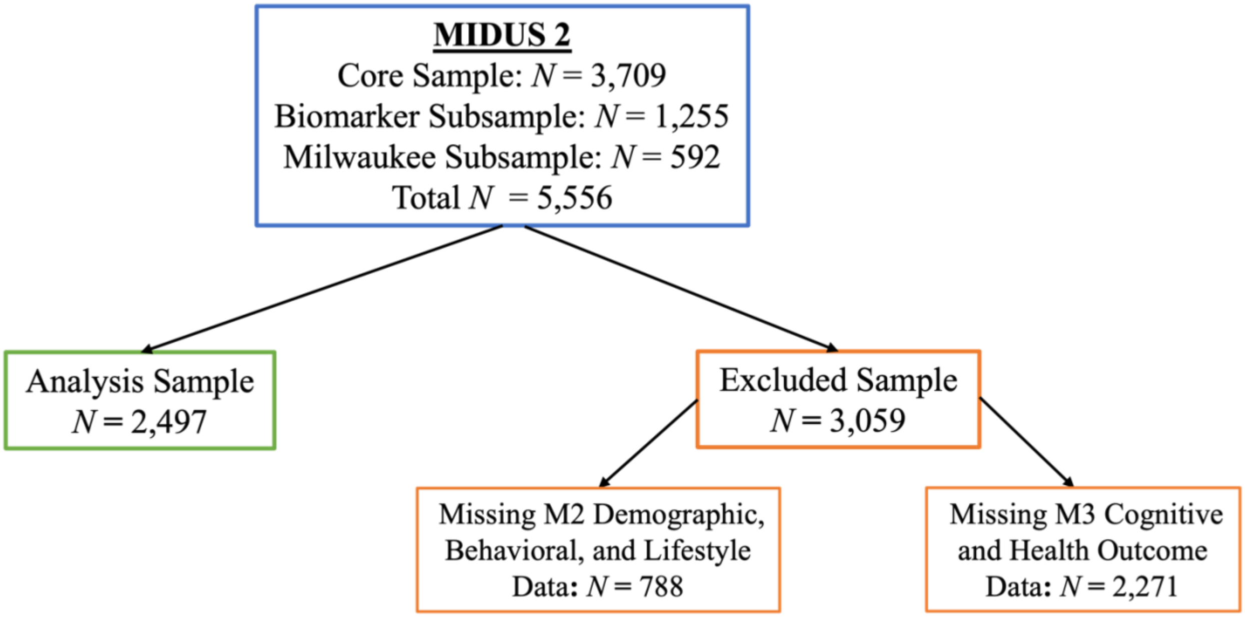

Supplemental Table 1

Comparison between Analysis Sample and Excluded Sample

**Variables**	**Study Sample** (*N* = 2,497) **Mean (*SD*)**	**Excluded Sample** (*N* = 3,059) **Mean (*SD*)**
M2 Age	54.85 (11.22)	55.18 (13.36)
M2 Sex	57% women, 43% men	52.5% women, 47.5% men***
M2 Race	88% White, 12% non-White	76% White, 24% non-White***
M2 Years of Education	14.50 (2.65)	13.63 (2.63)***
M2 Episodic Memory	.129 (.964)	-.162 (1.02)***

M3 Episodic Memory	-.030 (.992)	-.143 (1.05)***
M2 Executive Function	.184 (.930)	-.229 (1.04)***
M3 Executive Function	-.180 (.749)	-.354 (.939)***
M2 Functional Health	83.86 (23.5)	74.49 (29.3)***
M3 Functional Health	75.37 (28.5)	69.65 (31.0)***
M2 Chronic Conditions	2.60 (2.57)	3.21 (3.04)***
M3 Chronic Conditions	3.29 (2.78)	3.78 (3.21)***

Note. Functional Health ranged from 0 to 100. Chronic Conditions ranged from 0 to 30. ***Excluded sample descriptives are significantly different from study sample descriptives at $p < .001$.

Supplemental Table 2

M2 Demographic, Behavioral, and Lifestyle Covariate Descriptives

Covariates	Description	Minimum, Maximum	Mean (*SD*)
Age	Years	33, 83	54.85 (11.22)
Sex	Male, Female	1, 2	1.57 (.496)
Race	White, Non-White	1, 2	1.12 (.323)
Education	Years	6, 20	14.50 (2.65)
Smoking	Current smoker, Non-Smoker	1, 2	1.87 (.341)
Alcohol/Drug Problems	No, Yes	0, 1	.01 (.100)
Waist Circumference	Inches around the navel	-2.04, 4.60	-.025 (.942)
Physical Activity	Never, Several times a week or more	1, 6	3.36 (.764)

Supplemental Table 3

Regressions – Individual Psychosocial Factors and Cognition and Health Outcomes

	**M3 Episodic Memory**	**M3 Executive Function**	**M3 Functional Health**	**M3 Chronic Conditions**
**Predictors**	*b* (*SE*), *β*	*b* (*SE*), *β*	*b* (*SE*), *β*	*b* (*SE*), *β*
M2 Sense of Control	**.055 (.017), .054*****	.016 (.010), .020	**2.05 (.454), .070*****	**-1.70 (.046), -.059*****
M2 Purpose in Life	**.057 (.017), .055*****	.016 (.010), .021	**1.95 (.450), .066*****	-.046 (.046), -.016
M2 Social Support	**.092 (.036), .042****	**.058 (.021). .034****	**2.73 (.970), .043****	-.197 (.099), -.031

Note. For the purposes of brevity, we only included the regression coefficients for the individual psychosocial factors. However, these models included all covariates (age, sex, education, race, smoking, alcohol/drug, waist circumference, physical activity, and respective M2 cognition or health). M2 = MIDUS 2. M3 = MIDUS 3. *b* = unstandardized coefficient. *SE* = standard error. *β* = standardized coefficient. Significant regressions are bolded. ** $p < .01$. *** $p < .001$.

Supplemental Table 4

Regressions – Simultaneous Psychosocial Factors and Cognition and Health Outcomes

	**M3 Episodic Memory**	**M3 Executive Function**	**M3 Functional Health**	**M3 Chronic Conditions**
**Predictors**	*b* (*SE*), *β*	*b* (*SE*), *β*	*b* (*SE*), *β*	*b* (*SE*), *β*
M2 Sense of Control +	.031 (.021), .030	.005 (.012), .006	1.20 (.562), .041	**-.198 (.057), -.069*****
M2 Purpose in Life +	.032 (.021), .031	.004 (.012), .005	1.12 (.561), .038	.084 (.057), .029
M2 Social Support	.040 (.040), .018	.050 (.023), .030	.894 (1.06), .014	-.115 (.109), -.018

Note. For the purposes of brevity, we only included the regression coefficients for the simultaneously entered psychosocial factors. However, these models included all covariates. M2 = MIDUS 2. M3 = MIDUS 3. *b* = unstandardized coefficient. *SE* = standard error. *β* = standardized coefficient. Significant regressions are bolded. *** $p < .001$.
